# 20184 A Hypothesis-Driven Parametric Study of a Computational Dermal Replacement Model

**DOI:** 10.1017/cts.2021.517

**Published:** 2021-03-30

**Authors:** David Sohutskay, Adrian Buganza Tepole, Sherry Voytik-Harbin

**Affiliations:** 1 Purdue University Weldon School of Biomedical Engineering; 2 Purdue University Department of Mechanical Engineering

## Abstract

ABSTRACT IMPACT: This work will be used to improve the design of engineered dermal replacements that can be used to treat difficult-to-heal wounds such as burns or ulcers. OBJECTIVES/GOALS: Wounds of the skin are among the most common and costly medical problems experienced. Engineered dermal replacements have been developed to improve outcomes, but the optimal design features are unknown. Here we describe a hypothesis-driven study of scaffold parameters using a computational model of wound healing to simulate a variety of treatments. METHODS/STUDY POPULATION: The computational model, which was informed by animal data, was used to simulate cell, cytokine, and collagen density fields. There are reciprocal mechanobiological interactions between the cells and collagen that guide the wound healing process. We analyzed initial wound properties such as scaffold stiffness, microstructure, degradation, and wound geometry by running a one-at-a-time order-of-magnitude parameter change. We then conducted a derivative-based local sensitivity analysis for simulated experimental conditions and constructed a surrogate model of wound contraction using Gaussian process regression. RESULTS/ANTICIPATED RESULTS: We conducted finite element model simulations of scaffolds that varied in physical properties. A sensitivity analysis demonstrated that wound contraction was highly sensitive to collagen fiber stiffness and density. Wound contraction rate was also dependent on initial wound size and surface area. Collagen fiber orientation in the scaffolds affected contraction directionality and the orientation of the final wound area. A Gaussian process regression model was fit to the simulation results for use in rapid prototyping of scaffolds for design optimization. The Gaussian process model was able to reproduce the wound contracture for training and test cases. DISCUSSION/SIGNIFICANCE OF FINDINGS: This work further analyzes a computational model of wounds treated with collagen scaffold dermal replacements. The hypothesis driven analysis of the model suggested several key design features of scaffolds. The model surrogate will further be used for the purposes of prediction and optimization of tissue regeneration outcomes.

